# Foraging Behavior and Pollen Transport by Flower Visitors of the Madeira Island Endemic *Echium candicans*

**DOI:** 10.3390/insects12060488

**Published:** 2021-05-24

**Authors:** Fabiana Esposito, Ricardo Costa, Mário Boieiro

**Affiliations:** 1 Centre for Ecology, Evolution and Environmental Changes (cE3c), Faculty of Sciences, University of Lisbon, 1749-016 Lisbon, Portugal; rcosta47447@gmail.com; 2 Centre for Ecology, Evolution and Environmental Changes (cE3c), Azorean Biodiversity Group, University of Azores, 9700-042 Angra do Heroísmo, Portugal; mario.rc.boieiro@uac.pt

**Keywords:** pollination efficiency, pollen loads, visitation rates, island endemic, non-native bees

## Abstract

**Simple Summary:**

The successful conservation of many endangered island plants depends on the pollination services provided by animals. In this study, we identify the flower visitors of *Echium candicans*, a charismatic plant exclusive to the island of Madeira, and also evaluate their performance as pollinators by analyzing their behavior on the flowers and the pollen they transport on their body. We found that many different animals visit this plant’s flowers, from insects to reptiles, but bees were the most frequent visitors. Large bees visited more flowers and transported more pollen of *Echium candicans* compared to other pollinators, like butterflies and hoverflies. However, by visiting many flowers in the same plant large bees might contribute to inbreeding, whilst the other animals visited fewer flowers in each plant favoring outcrossing. We conclude that the different flower visitors of *Echium candicans* provide complementary services as pollinators and highlight the importance of having diverse communities of pollinators to ensure successful pollination in many island plants.

**Abstract:**

The study of flower visitor behavior and pollen transport dynamics within and between plants can be of great importance, especially for threatened or rare plant species. In this work, we aim to assess the flower visitor assemblage of the Madeiran endemic *Echium candicans* and evaluate the performance of the most common visitors through the analysis of their foraging behavior and pollen loads. The flower visitor assemblage of *E. candicans* is diverse, including several insect groups and the endemic lizard *Teira dugesii*, but bees are the most common visitors. In general, large bees (*Amegilla quadrifasciata*, *Apis mellifera*, and *Bombus* spp.) had the highest average visitation rates (>18 flowers/min) and their pollen loads had higher percentages of homospecific pollen (>66%) when compared with butterflies and hoverflies. The honeybee (*Apis mellifera*) and two bumblebees (*Bombus terrestris* and *B. ruderatus*) were the most efficient flower visitors of *E. candicans*, but their foraging behavior seems to favor geitonogamy. Other visitors, such as butterflies and the small bee *Lasioglossum wollastoni*, may have a complementary role to the honeybee and bumblebee species, as their high mobility is associated with fewer flower visits on each plant and may promote xenogamy. Two non-native bees (*A. mellifera* and *B. ruderatus*) are important flower visitors of *E. candicans* and may contribute mostly to self-pollination rendering the endemic plant more vulnerable to inbreeding effects.

## 1. Introduction

Most flowering plants rely on animals for pollination [1] and a single plant may be visited by a wide taxonomic diversity of flower visitors including both vertebrates and invertebrates, but, in general, most visitors are insects such as ants, bees, beetles, butterflies, flies, moths and wasps [2]. However, not all flower visitors are pollinators since some animals may use the floral resources without providing a pollination service [3,4]. Pollinators are not equally efficient and effective in providing the pollination service [5,6]. The size, morphology, and hairiness of specific anatomical structures (e.g., mouthparts, legs) may strongly influence pollen collection, transport, and transfer between flowers [7,8,9,10]. For example, a recent experimental study on the mechanics of pollen removal and deposition showed that proboscis width was the critical factor determining pollination effectiveness [11]. Pollinator behavior, in particular flower visitation rates and the movement patterns within and between conspecific plants, may strongly drive the differences in pollination efficiency by the different flower visitors [12,13,14]. 

Several metrics have been proposed to investigate differences in pollinator performance. These metrics rely on the assessment of two independent components [15,16,17]: a quantitative component, usually associated with flower visitation frequency [18,19], and a qualitative component that evaluates the ability of flower visitors to successfully deliver pollen grains to conspecific stigmas [2,19,20]. The use of indirect methods may lead to less accurate results that should be interpreted with caution but presents the advantage in allowing to overcome the major difficulties involved in the study of large and taxonomic diverse assemblages of flower visitors as is the case of many natural pollination systems.

During the last few centuries, oceanic island biodiversity has been severely affected by human-mediated changes with many species being lost and many others now lying vulnerable to extinction [21,22]. Several studies have stressed that the conservation management of these threatened island endemic species should not strictly follow a traditional species-based approach but also value the importance of ecological interactions [23,24] as their survival may be influenced by the presence of other groups of organisms with which they interact [25]. In fact, pollination and seed production often represent the most vulnerable stages of many threatened plants, therefore it is critical to have good knowledge on the diversity of flower visitors, their effectiveness as pollinators, and the dynamics of pollen transfer for their effective conservation [14,26,27]. 

Previous investigations on the interactions between plants and their insect pollinators in Macaronesia have mostly been carried out in the Canary Islands, e.g., [14,27,28], with several other contributions from the Azores archipelago [28,29,30,31]. In the Madeiran archipelago, much fewer studies were carried out, usually being taxonomically biased and consisting mostly of lists of species associations resulting from unstandardized sampling [28,29,32,33,34,35]. Following the study of pollination networks on five oceanic islands (including Flores in the Azores), Olesen et al. [36] identified the prevalence of endemic species with a very wide pollination niche, which were coined as super generalists. This island phenomenon seems to be a consequence of the low species diversity and low interspecific competition in these ecosystems [36], but further studies are needed to identify functional and phylogenetic correlates of interaction generalization. Several Macaronesian endemic plants, including representatives of genus *Echium*, can be classified as generalists since they interact with a much higher number of flower visitor species than the other co-occurring plants [29]. 

The present biodiversity crisis on oceanic islands is driven to some extent by the high rates of species introductions [37]. Since the integration of mutualistic non-native species in native communities is usually mediated by super generalist endemic species [36,37,38], it will be critical to monitor island pollination networks and evaluate how the visitation behavior of non-native pollinators may affect native plants reproduction.

In this work, we investigate the diurnal community of flower visitors of the Madeira island endemic *Echium candicans*. We aim to characterize the assemblage of flower visitor species and evaluate flower visitation rates and the transport of homospecific pollen by the most frequent insect visitor species. We are particularly interested in: (1) identifying the insect groups and species that are responsible for most visits to the flowers of *E. candicans*; (2) assessing visit duration time and movement between flowers and inflorescences by the most frequent insect visitors; and (3) analyzing the purity of pollen loads (i.e., homospecific vs. heterospecific pollen) carried by the most frequent insect visitors. Finally, we discuss the potential impact of two non-native bee species (*Apis mellifera* and *Bombus ruderatus*) on the reproduction of the island endemic plant.

## 2. Materials and Methods

### 2.1. Study Species

**Figure 1:**
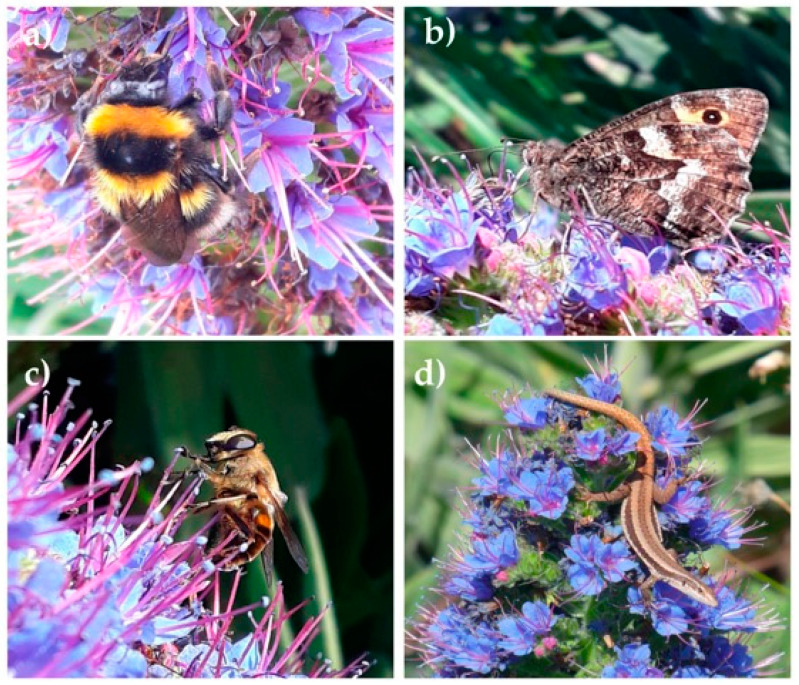
Some frequent flower visitors of *Echium candicans* at Pico do Arieiro: (**a**) the non-native *Bombus ruderatus*; (**b**) the endemic *Hipparchia maderensis*; (**c**) the native *Eristalis tenax*, and (**d**) the endemic lizard *Teira dugesii*.

*Echium candicans* L.f. (Boraginaceae) is a shrub that can grow up to 2 m on forest-cliff habitats and terraces above 800 m on the cloud zone of the Madeira Island [39,40]. Typically, *E. candicans* is found in open forest border areas, as well as on rocky cliffs [41,42]. This Madeiran endemic plant is currently protected by the Habitats Directive (Annex II and IV) and classified as Data Deficient by the IUCN [40]. The main threats to its survival are the loss of habitat due to human activities and wildfires, and the spread of invasive plant species [40,41,42]. This plant is characterized by large cylindrical inflorescences (Figure 1) with many blue funnel-shaped protandrous flowers, which might be an adaptation to reduce autogamy [39,40,41,42,43]. The flowering period lasts from April to August [44] depending on the altitude of the populations. *Echium candicans* is a suitable model to study differences in pollinator foraging behavior since it produces a huge number of flowers that provide both nectar and pollen, attracting a wide variety of flower visitor groups.

### 2.2. Study Area

Flower visitors were surveyed from two populations of *E. candicans*, located near Pico do Arieiro in Madeira Island, one at 1500 m (32°43′08″ N: 16°54′31″ W) and the other at 1800 m (32°44′ N; 16°55′47″ W) above sea level. The study area is part of a Special Conservation Area (Habitats Directive; PTMAD0002—Central Mountainous Massif), enclosed in the Madeira Natural Park [45]. *Echium candicans* was common in both study sites, where it co-occurs with other endemics, like *Erica maderensis* (Ericaceae), *Vaccinium padifolium* (Ericaceae), *Melanoselinum decipiens* (Apiaceae), *Teline maderensis* (Fabaceae), and *Argyranthemum pinnatifidum* (Asteraceae) [39,40,41,42]. This area was severely affected by goat grazing until its ban by the end of the last century [45]. In recent years wildfires and the spread of invasive plants, such as the common broom *Cytisus scoparius* (Fabaceae) [4], one of the worst invasive species in the Macaronesian archipelagos [46], changed the landscape and had a negative impact on the vegetation structure and composition.

### 2.3. Sampling Flower Visitors and Their Foraging Behavior

Sampling of the flower visitor species of *E. candicans* was performed from early July to the beginning of August 2018 and consisted of 10 min observation periods of animal visitation to the flowers of a focal plant. The observations on each plant were made during the peak of flower-visiting activity (from 10:00–16:00), under favorable climatic conditions. Overall, 24 h of observations were performed on different plant individuals (*n* = 24) to assess the flower-visitor assemblage of *E. candicans*. We registered the lowest taxonomic identity possible of each visitor species after they first touched the reproductive parts of a flower. Many flower visitors were identified to species level on the spot, while others were collected with a sweeping net for identification at the stereomicroscope (Olympus SZX7) in the laboratory using taxonomic literature, e.g., [47,48,49,50,51,52]. Insect sampling was authorized by the Madeiran legal authorities (Instituto das Florestas e Conservação da Natureza, IFCN) and all captured specimens were preserved in ethanol (70%) and deposited in the entomological collection of the Laboratory of Entomology of the Faculty of Sciences (University of Lisbon). 

Sampling of the foraging behavior of flower visitors was carried out during July 2018, with additional sampling in June 2019. The most common flower visitor species of *E. candicans* were selected for this study, which included five bees (*Apis mellifera*, *Amegilla quadrifasciata*, *Bombus ruderatus*, *B. terrestris*, and *Lasioglossum wollastoni*), two butterflies (*Colias croceus* and *Hipparchia maderensis*), and two hoverflies (*Eristalis tenax* and *Scaeva pyrastri*). We also performed foraging behavior observations of the endemic Madeiran lizard *Teira dugesii*, a less frequent, but locally important, flower visitor. During each observation period, we recorded the duration of the visits to the plant (up to 10 min), and the number of inflorescences and flowers visited. Up to twenty individuals of each of the ten selected species were tracked during their visit to the flowers of randomly chosen *E. candicans* plants. Overall, we recorded the activity of 198 individual visitors with a total of 665 min of observation. 

### 2.4. Pollen Transport by Flower Visitors

To estimate the potential contribution of insect visitors to the pollination of *E. candicans*, 10 individuals of each insect species selected for the behavioral study were collected during field sampling, preserved dry, and later analyzed in the laboratory. First, we examined the insect body to identify the areas that are suitable for pollen transfer, and then, before proceeding with pollen analysis, we removed the hind legs of all large bees since the pollen storage there is packed and unavailable for the pollination service [53]. Pollen loads were analyzed following a modified version of the method proposed by MacGillivray (1987) [54]. For most insect specimens, pollen collection was achieved by placing the insect body in 1.5 mL Eppendorf tubes and by adding ethanol (70%). However, for butterflies, to avoid contamination with scales, pollen was gently removed from the body to slides (with a drop of ethanol) using a small brush, and then the solution was sealed for microscopic analysis. The Eppendorf tubes were manually agitated by hand for 2 min to displace the pollen grains from the insect body, and after removing the specimens, the solutions were centrifuged at 10,000 rpm for 15 min. The resulting pollen suspension was dried for 24 h at room temperature. Subsequently, a drop of pollen pellet was transferred to slides and analyzed at a microscope (Leica CME). We analyzed the pollen loads from 10 individuals of each selected insect species by identifying and counting homospecific and heterospecific pollen grains from 10 randomly selected fields in each slide. Then, we calculated the percentage of homospecific pollen grains transported by each insect species (number of *E. candicans* pollen grains divided by total pollen grains counted) by averaging the results from slide fields and specimens. 

### 2.5. Statistical Analysis 

To assess differences in the foraging behavior of the different flower visitors, we first calculated the average visitation rate of each species by dividing the number of flowers visited by visit duration time of the different observations from conspecific visitors [55]. Then, we assessed the interspecific differences in visit duration, the number of inflorescences visited, the number of flowers visited, and the visitation rate between flower visitor species. We first tested if the variables could be analyzed using a parametric variance test, but since they did not meet the assumptions of normality and homoscedasticity, we used Kruskal-Wallis non-parametric tests to check for differences between species in the four selected variables. Statistically significant results (*p* < 0.05) were followed by post-hoc Dunn’s tests to determine which species differed between each other in specific foraging behavior variables. Intra- and interspecific differences in the percentage of *E. candicans* pollen grains (homospecific) carried by the selected flower visitors were assessed using Wilcoxon/Mann-Whitney tests. All statistical analyses were performed using R statistical software, version 3.6.0 [56]. 

## 3. Results

### 3.1. The Assemblage of Flower Visitors of Echium candicans 

We observed a total of 5612 flower visitors from 51 different morphospecies, most of which (34) were identified at the species level (Table S1). Among the identified taxa, 25% are endemic, 66% are native non-endemic (from now on referred to as native) and only 9% are non-native to Madeira Island [52,57]. The flower visitors were mostly insects belonging to three species-rich orders (Hymenoptera, Diptera, and Lepidoptera), with the most common visitors being bees (55% of all observations), butterflies (20%), and hoverflies (9%) (Table 1). We selected the most common flower visitors of *E. candicans* to further investigate their foraging behavior and assess their role as pollen vectors. The Madeiran lizard *Teira dugesii*, which was observed lapping the nectar of *E. candicans* flowers, was also included in the flower visitation study. Altogether, the ten selected species accounted for 81% of the visits to the flowers of the study plant.

### 3.2. Foraging Behavior of Flower Visitors

**Figure 2:**
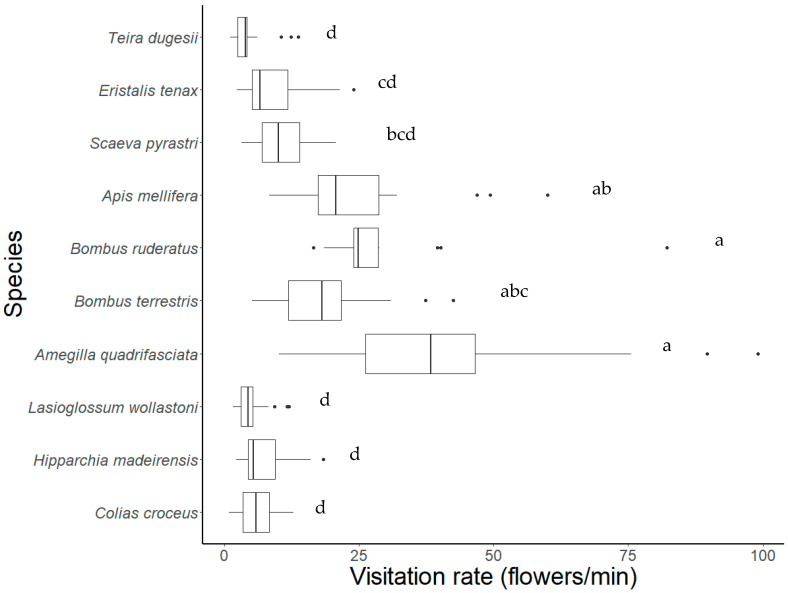
Visitation rates of the most common flower visitors of *E. candicans*. Twenty individuals/species were sampled except for *L. wollastoni* (*n* = 18). Data are presented as boxplots with descriptive values (minimum, first quartile, median, third quartile, maximum) and outliers. Letters designate groups of species that are statistically similar (*p* < 0.05). Visitors belong to four animal groups: butterflies, bees, hoverflies, and the lizard.

The analysis of the different components of the foraging behavior of flower visitors showed significant differences between species. The visit duration was larger for the hoverflies (particularly *S. pyrastri*) when compared with most bee species (e.g., *A. quadrifasciata*) and the butterfly *C. croceus* (Table 2). The butterfly *H. maderensis* and the lizard *T. dugesii* visited a significantly lower number of inflorescences than the large bees (*A. quadrifasciata*, *A. mellifera*, and *Bombus* spp.) and the hoverfly *S. pyrastri*, which explored the complex plant architecture of *E. candicans* by foraging on several inflorescences. These same species also visited a much larger number of flowers than the other insect species and, curiously, the endemic lizards probed few flowers during their visits. The analysis of interspecific differences in visitation rates clearly highlights the large bees as the most efficient flower visitors followed by the hoverflies (Figure 2). In contrast, the small bee (*L. wollastoni*) and the endemic lizard visited a very low number of flowers per time unit since the former showed a more elaborated behavior when searching for pollen while the latter alternated active foraging with resting periods.

### 3.3. Pollen Transport by Flower Visitors

**Figure 3:**
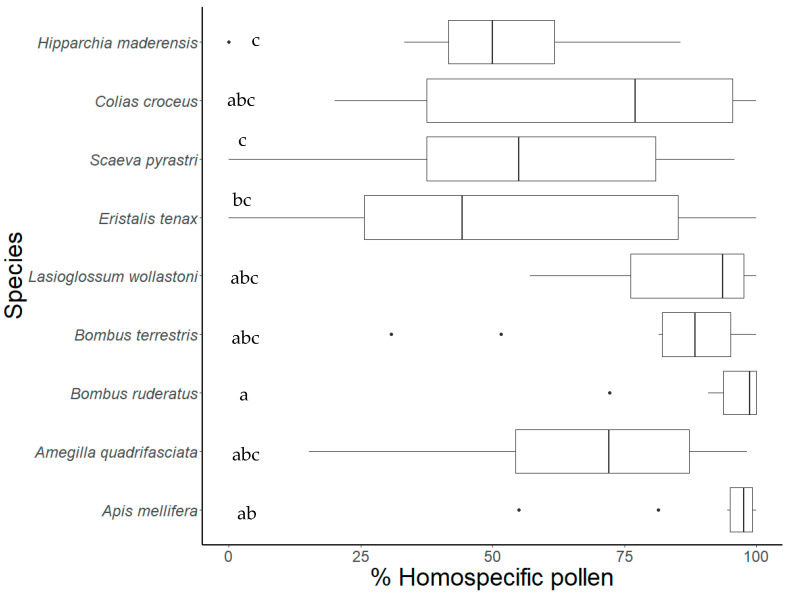
Percentage of homospecific pollen collected by the most common flower visitor species of *E. candicans*. Ten samples from 10 individuals/species. Data are presented as boxplots with descriptive values (minimum, first quartile, median, third quartile, maximum) and outliers. Letters designate groups of species that are statistically similar (*p* < 0.05). Visitors belong to three animal groups: bees, hoverflies, and butterflies.

The observation of pollen grains on insects showed that all species carried pollen of *E. candicans* on their body. The bees, except *A. quadrifasciata*, carried mostly homospecific pollen on their bodies (e.g., >80%) compared to the other insect species (Figure 3; Table S2). In butterflies and hoverflies, the transport of homospecific pollen was on average lower than 70%. Significant differences in homospecific pollen transport were found between the two non-native bee species (*A. mellifera* and *B. ruderatus*) and both the native *S. pyrastri* and the endemic *H. maderensis* (all *p* < 0.05). Furthermore, homospecific pollen transport by *B. ruderatus* also differed from that carried out by the hoverfly *Eristalis tenax* (*p* = 0.039) (Figure 3). All bee species and the butterfly *C. croceus* showed significant differences in the percentage of homospecific versus heterospecific pollen transported in their bodies (all *p* < 0.05), contrasting with the hoverflies and the butterfly *H. maderensis*, which showed no differences between the percentage of pollen types (Table S2).

**Figure 4:**
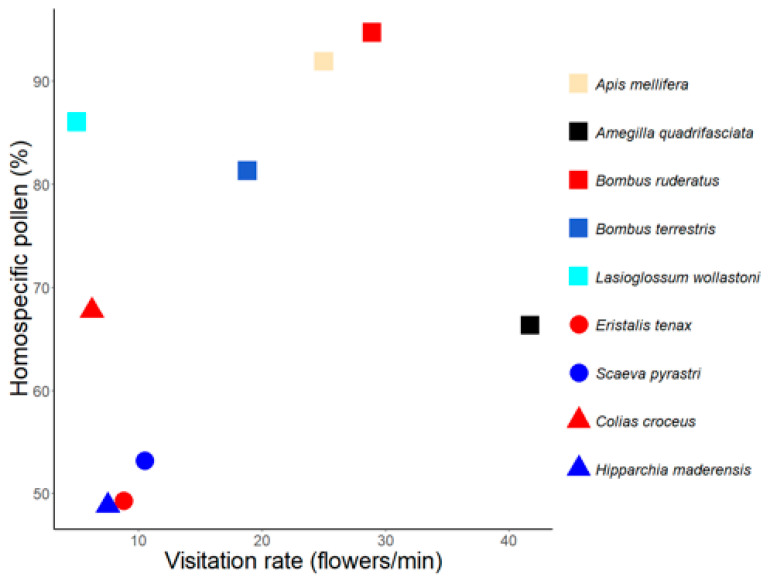
Average visitation rate and percentage of homospecific pollen found on the most common flower visitors of *E. candicans*. Visitors belong to three animal groups: bees (squares), hoverflies (circles), and butterflies (triangles).

Interestingly, most of the insect species with higher visitation rates, particularly bumblebees and the honeybee, also transported high levels of homospecific pollen (Figure 4). The endemic bee *A. quadrifasciata*, despite being a frequent flower visitor of *E. candicans*, presented pollen loads with moderate levels of homospecific pollen. Among the taxonomic diverse assemblage of insects with lower visitation rates there was not an evident pattern on homospecific pollen load transport since some species carried high levels (e.g., *L. wollastoni*) while others very low, not even reaching 50% (e.g., *H. maderensis*).

## 4. Discussion

### 4.1. The Flower Visitors of Echium candicans

The flowers of the Madeira island endemic *E. candicans* are visited by different animal groups, including butterflies, hoverflies, and lizards, but overall, bees were the most commonly observed flower visitors (Table 1). Bees have been reported as frequent flower visitors of *Echium* species, including the Madeiran and Canarian endemics [27,28,33,34,58,59] as many plants of this genus are considered important providers of food resources (pollen and nectar) to flower visitors [33,34,60]. Our results reinforce this finding but, contrary to previous studies on the Canarian *Echium* [27,58,59,61], they highlight the high diversity and frequency of hoverflies and butterflies as flower visitors. These two insect groups are important pollinators of many plants and, due to their specific anatomy and foraging behavior, may provide a complementary service to that of bee species, e.g., [62]. Particularly interesting were also the observations of the Madeiran lizard *Teira dugesii*, which was detected as a frequent flower visitor of *E. candicans* at the high altitudes of Pico do Arieiro (Figure 1). Its occurrence was, however, more local and seemed associated with rocky areas, where the reptiles can find shelter in crevices and thermoregulate more effectively. The flower-visiting behavior of this island endemic reptile was among the first to be recorded, and since then it is a well-known flower visitor of many coastal plants in Madeira, including the other endemic species of *Echium* [63,64,65].

The assemblage of flower visitors of *E. candicans* is taxonomically and functionally diverse and most species are endemic or non-endemic native [57]. However, it should be noted that two non-native visitor species, the honeybee, and the bumblebee *Bombus ruderatus*, were responsible for a third of the visits to the endemic plant. Further studies are needed to assess the ecological impact of these non-native species on the local pollinator communities and the reproduction of Madeiran vulnerable endemic plants.

### 4.2. Foraging Behavior and Pollen Loads of Flower Visitors 

In this study, we determined the quantitative (visitation) and qualitative (pollen load purity) components of the pollination service by the most common flower visitors of *E. candicans*. In general, large bees (*A. quadrifasciata*, *A. mellifera*, and *Bombus* spp.) had higher visitation rates to flowers and visited more inflorescences in each plant than the other animal groups. This finding agrees with previous studies on flower visitation [27,66,67] where bees were found to be the most efficient visitors since they moved quickly between flowers and presented lower flower handling times. 

Differences in flower visitation between large bees and the other animals are a consequence of the behavioral and anatomical differences between them (e.g., nectar extraction apparatus), but also reflect differences in resource use [55]. For example, both the small bee *L. wolllastoni* and the hoverfly *S. pyrastri* occasionally visited the flowers just in search of pollen (not nectar), thus presenting higher flower handling times. The butterfly *Hipparchia maderensis* had low visitation rates as it took much longer periods probing and feeding on flowers from a few inflorescences. Therefore, considering their high visitation rates, we assume that large bees could provide a more efficient pollination service when compared with the other animal groups. 

Several studies stress the role of bees as key pollinators by emphasizing their performance in visiting flowers, e.g., [27,55]. For instance, in a comparative experimental study, Jauker et al. [68] found that hoverflies were less efficient pollinators than bees since they had longer flower handling time and their visitation rate was density-independent. 

The assessment of pollen loads on the insect bodies showed that all flower visitor species transported pollen grains of *E. candicans*, but bees carried the highest percentages of homospecific pollen. On average, pollen load purity carried by bees varied between 66–95%, with most individuals of *Apis mellifera* and *Bombus ruderatus* carrying over 90% of *E. candicans* pollen. Many bee species exhibit high plant fidelity and transport significant pollen loads, which coupled with high flower visitation rates, make them amongst the most important contributors for pollination effectiveness [2,69]. Nevertheless, most flower visitors of *E. candicans* are opportunistic nectar and pollen foragers, being known to have a polylectic behavior, e.g., [34,52,59,60]. 

The bee *A. quadrifasciata* transported a lower percentage of homospecific pollen compared to the other bees, suggesting a nonlinear association between visitation frequency and pollination effectiveness, e.g., [11,55]. The hoverflies and the butterfly *Colias croceus*, which are known to carry pollen from a wide variety of plant species, seem to play a minor role in the pollination of *E. candicans* since they visited the plants less often than bees and presented the lowest percentages of *E. candicans* pollen on their bodies (nearly 50%, on average). However, several studies have stressed the importance of the complementary role of flower visitor species/groups in contributing to pollination efficiency by simultaneously considering flower visitor attributes, plant breeding systems, and reproductive traits, jointly with the assessment of plant reproductive output [14,70]. For instance, Barrios et al. [11] showed that visitation frequency was not a good predictor of pollination efficiency since the long-tongued bees were in fact more effective in pollen transfer between the tubular flowers of *Angadenia berteroi* than the most common insect visitors. More recently, Jaca et al. [14] also found that differences in pollination behavior of diurnal and nocturnal flower visitors of the Canarian endemic *Echium simplex* led to the complementary reproductive success of the different inflorescence sections of this plant. 

The role of honeybees as pollinators has been highlighted from both natural and agricultural systems, but some studies stress that their contribution to pollination comes mostly from the high visitation rates, not from high pollen transfer efficacy [71]. Despite several authors considering flower visitation frequency a more important parameter than effectiveness on a per-visit basis when assessing species contribution to pollination [2,13,20,72], detrimental effects of honeybee pollination on plant reproductive success have often been reported since this species may contribute mostly for selfing (e.g., geitonogamous pollination), not to outcrossing [73,74,75]. In fact, negative consequences of inbreeding as a result of honeybee pollination have been reported from several plants and may include lower fruit and seed set, lower seed viability, late-acting effects on offspring, and even changes in gene flow that may affect the genetic structure of plant populations [59,74,75,76]. Thus, several authors argue that is crucial to maintain the diversity of flower visitors since they usually fulfill different roles in plant pollination, with frequent visitors, in general, pollinating a higher number of flowers while other visitors may deliver better quality pollen, e.g., [18,55,77,78]. To confirm this hypothesis for the flower-visiting community of *E. candicans*, future studies should evaluate the individual plant reproductive success (e.g., seed set) associated with single visits to virgin flowers [17]. This being a very technical and time-consuming task, it should be applied to a small subset of the flower visitor species. 

## 5. Conclusions

The knowledge on pollen dynamics and pollinator efficiency is crucial for the conservation management of island endemic plants that depend on animal pollination for reproduction, as is the case of our studied Madeira *Echium candicans.* The floral traits and the high amount of resources (pollen and nectar) produced by this endemic plant attract a wide diversity of visitors from different insect groups, being a key species on mountainous and open forest areas by supporting the populations of many native species. 

The most frequent flower visitors of *E. candicans* showed significant differences in visitation and pollen transport behavior that may suggest a complementary pollination service, with some species (e.g., large bees) favoring geitonogamy while others (e.g., butterflies, small bee) promoting xenogamy. An alarming result of our study was the finding that two non-native bee species, *A. mellifera* and *B. ruderatus*, are amongst the most frequent visitors of *E. candicans*, counting a third of the total visits. Despite playing an effective pollination service for many plant species, both bee species move less between conspecific plants than other flower visitors, thus increasing the probabilities of self-pollination and the potential negative consequences of inbreeding for Madeira island plant populations. In addition, these non-native mutualists (particularly the honeybee), when present in high abundance, may severely impact the native pollination networks by outcompeting native flower visitors leading to a reduction in both local insect species diversity and plant-pollinator interaction links [58,59,79,80,81]. 

Future studies are needed to understand the impact of these non-native pollinators on the reproductive success of threatened Madeira island endemic plants and the abundance and diversity of native flower visitors, particularly on endemic bees. It is also critical to identify and analyze the changes in the structure and functioning of pollination networks caused by these two non-native bees to better understand, foresee and manage their impact on Madeira native biodiversity and plant-pollinator interactions.

## Figures and Tables

**Table 1:** The main groups and species of flower visitors observed on *Echium candicans* at Pico do Arieiro, their nativeness, and the overall number of visits to plants in the two study sites. The most common visitor species (highlighted in bold) were selected for the behavioral study.

Group	Species	Observations	Nativeness
Bees	***Amegilla quadrifasciata maderae***	316	Endemic
	*Andrena wollastoni*	110	Endemic
	***Apis mellifera***	578	Non-native
	***Bombus ruderatus***	1278	Non-native
	***Bombus terrestris***	310	Native
	*Halictus frontalis*	11	Endemic
	*Hoplitis acuticornis*	2	Native
	***Lasioglossum wollastoni***	487	Endemic
Butterflies	***Colias croceus***	94	Native
	*Danaus plexippus*	1	Native
	***Hipparchia maderensis***	992	Endemic
	*Lampides boeticus*	3	Native
	*Leptotes pirithous*	3	Native
	*Lycaena phlaeas*	9	Native
	*Pieris rapae*	1	Non-native
	*Vanessa atalanta*	1	Native
	*Vanessa cardui*	8	Native
Hoverflies	***Eristalis tenax***	216	Native
	*Eupeodes* spp.	46	Native
	*Paragus coadunatus*	2	Native
	*Scaeva albomaculata*	1	Native
	***Scaeva pyrastri***	182	Native
	*Scaeva selenitica*	21	Native
	*Sphaerophoria rueppellii*	1	Native
	*Sphaerophoria scripta*	37	Native
	*Xanthandrus babyssa*	1	Endemic
Lizards	***Teira dugesii***	92	Endemic

**Table 2:** Differences in components of foraging behavior between flower visitors of *E. candicans*. Twenty individuals/species were sampled except for *L. wollastoni* (*n* = 18). Data are presented as mean ± SD. Letters designate groups of species that are statistically similar (*p* < 0.05) to a particular behavior characteristic.

Group	Species	Visitation Time (s)	Number of Inflorescences Visited	Number of Flowers Visited
Bees	*Amegilla quadrifasciata*	118 ± 100 ^a^	11.7 ± 8.9 ^a^	79.8 ± 71.1 ^a^
	*Apis mellifera*	237 ± 193 ^ab^	10.1 ± 7.3 ^ab^	87.3 ± 68.9 ^a^
	*Bombus ruderatus*	149 ± 100 ^ab^	7.5 ± 5.2 ^abc^	68.1 ± 45.4 ^a^
	*Bombus terrestris*	216 ± 177 ^ab^	6.7 ± 5.8 ^abc^	66.1 ± 60.6 ^a^
	*Lasioglossum wollastoni*	150 ± 129 ^ab^	4.1 ± 3.7 ^bcd^	9.8 ± 9.3 ^b^
Butterflies	*Colias croceus*	129 ± 120 ^a^	3.4 ± 2.4 ^cd^	9.6 ± 8.5 ^b^
	*Hipparchia maderensis*	248 ± 207 ^ab^	2.1 ± 1.2 ^d^	23.8 ± 18.7 ^ab^
Hoverflies	*Eristalis tenax*	292 ± 244 ^ab^	4.5 ± 2.4 ^abcd^	31.1 ± 26.3 ^ab^
	*Scaeva pyrastri*	328 ± 206 ^b^	7.2 ± 4.4 ^abc^	45.9 ± 22.0 ^a^
Lizards	*Teira dugesii*	142 ± 114 ^ab^	2.0 ± 1.3 ^d^	8.1 ± 6.0 ^b^

## Data Availability

All relevant data are within the paper and in Supplementary Materials.

## Supplementary Materials

The following are available online at https://www.mdpi.com/article/10.3390/insects12060488/s1, Table S1: Number of observations of flower visitor species/morphospecies of different animal groups on *Echium candicans*; Table S2: Percentage of homospecific and heterospecific pollen found on the most common flower visitors of *E. candicans* (* marks the species with significant differences between pollen types). Data are presented as mean ± SD.
