# Supplementary material for: Foraging Behavior and Pollen Transport by Flower Visitors of the Madeira Island Endemic Echium candicans
Source: Insects. 2021 May 24;12(6):488. doi: 10.3390/insects12060488 (PMC8225069; doi:10.3390/insects12060488)
Supplement: Supplementary file 1 [file insects-12-00488-s001.zip › Supplementary Materials - Table S1.pdf]

Supplementary Materials

Table S1 Number of observations of flower visitor species/morphospecies of different animal groups on *Echium candicans*.

Order	Family	Species/Morphospecies	Number of observations
Blattodea	-	*Gen sp1*	2
Coleoptera	Melyridae	*Gen sp1*	5
Coleoptera	Melyridae	*Psilothryx illustris*	63
Coleoptera	Nitidulidae	*Gen sp1*	3
Diptera	Anthomyiidae	*Gen sp1*	8
Diptera	Calliphoridae	*Calliphora* sp1	26
Diptera	Calliphoridae	*Gen sp1*	2
Diptera	Calliphoridae	*Stomorhina lunata*	6
Diptera	Conopidae	*Thecophora* sp1	14
Diptera	Lauxaniidae	*Gen sp1*	5
Diptera	Muscidae	*Gen sp1*	28
Diptera	Sarcophagidae	*Gen sp1*	7
Diptera	Sarcophagidae	*Sarcophaga* sp1	2
Diptera	Sepsidae	*Gen sp1*	9
Diptera	Syrphidae	*Eristalis tenax*	216
Diptera	Syrphidae	*Eupeodes* sp1	46
Diptera	Syrphidae	*Paragus coadunatus*	2
Diptera	Syrphidae	*Scaeva albomaculata*	1
Diptera	Syrphidae	*Scaeva pyrastri*	182
Diptera	Syrphidae	*Scaeva selenitica*	21
Diptera	Syrphidae	*Sphaerophoria rueppellii*	1
Diptera	Syrphidae	*Sphaerophoria scripta*	37
Diptera	Syrphidae	*Xanthandrus babyssa*	1
Hymenoptera	Andrenidae	*Andrena wollastoni*	110
Hymenoptera	Apidae	*Amegilla quadrifasciata*	316
Hymenoptera	Apidae	*Apis mellifera*	578
Hymenoptera	Apidae	*Bombus ruderatus*	1278
Hymenoptera	Apidae	*Bombus terrestris*	310
Hymenoptera	Crabronidae	*Gen sp1*	27
Hymenoptera	Formicidae	*Lasius grandis*	279
Hymenoptera	Formicidae	*Tapinoma madeirense*	70
Hymenoptera	Halictidae	*Halictus frontalis*	11
Hymenoptera	Halictidae	*Lasioglossum wollastoni*	487
Hymenoptera	Ichneumonidae	*Gen sp1*	29
Hymenoptera	Megachilidae	*Hoplitis acuticornis*	2
Hymenoptera	Pompilidae	*Gen sp1*	23
Hymenoptera	Sphecidae	*Gen sp1*	16
Hymenoptera	Sphecidae	*Podalonia* sp1	33

Hymenoptera	Vespidae	*Ancistrocerus* sp1	3
Hymenoptera	Vespidae	*Polistes dominulus*	144
Lepidoptera	Lycaenidae	*Lampides boeticus*	3
Lepidoptera	Lycaenidae	*Leptotes pirithous*	3
Lepidoptera	Lycaenidae	*Lycaena phlaeas*	9
Lepidoptera	Nymphalidae	*Danaus plexippus*	1
Lepidoptera	Nymphalidae	*Hipparchia madeirensis*	992
Lepidoptera	Nymphalidae	*Vanessa atalanta*	1
Lepidoptera	Nymphalidae	*Vanessa cardui*	8
Lepidoptera	Pieridae	*Colias croceus*	94
Lepidoptera	Pieridae	*Pieris rapae*	1
Lepidoptera	Sphingidae	*Macroglossum stellatarum*	3
Squamata	Lacertidae	*Teira dugesii*	92
