# Supplementary material for: Foraging Behavior and Pollen Transport by Flower Visitors of the Madeira Island Endemic Echium candicans
Source: Insects. 2021 May 24;12(6):488. doi: 10.3390/insects12060488 (PMC8225069; doi:10.3390/insects12060488)
Supplement: Supplementary file 1 [file insects-12-00488-s001.zip › Supplementary Materials - Table S2.pdf]

Supplementary Materials

Table S2 Percentage of homospecific and heterospecific pollen found on the most common flower visitors of *E. candicans* (* marks the species with significant differences between pollen types). Data are presented as mean±SD.

**Species**	**Homospecific pollen (%)**	**Heterospecific pollen (%)**
Apis mellifera *	91.9 ± 14.0	8.1 ± 14.0
Amegilla quadrifasciata *	66.3 ± 27.7	33.7 ± 27.7
Bombus ruderatus *	94.7 ± 9.0	5.3 ± 9.0
Bombus terrestris *	81.3 ± 22.5	18.7 ± 22.5
Lasioglossum wollastoni *	86.0 ± 15.9	14.0 ± 15.9
Eristalis tenax	49.3 ± 37.9	50.7 ± 37.9
Scaeva pyrastri	53.2 ± 33.6	46.8 ± 33.6
Colias croceus *	67.7 ± 32.1	32.3 ± 32.1
Hipparchia madeirensis	48.8 ± 25.2	51.2 ± 25.2
